# NCLs and ER: A stressful relationship

**DOI:** 10.1016/j.bbadis.2017.04.003

**Published:** 2017-06

**Authors:** Davide Marotta, Elisa Tinelli, Sara E. Mole

**Affiliations:** a MRC Laboratory for Molecular Cell Biology, University College London, Gower Street, London WC1E 6BT, United Kingdom; b The Institute of Cancer Research, 15 Cotswold Road, London SM2 5NG, United Kingdom; c Department of Genetics, Evolution and Environment, University College London, Gower Street, London WC1E 6BT; d UCL GOS Institute of Child Health, 30 Guilford Street, London WC1N 1EH, United Kingdom

**Keywords:** Batten disease, CLN1, CLN3, CLN6, CLN8, ER stress

## Abstract

The Neuronal Ceroid Lipofuscinoses (NCLs, Batten disease) are a group of inherited neurodegenerative disorders with variable age of onset, characterized by the lysosomal accumulation of autofluorescent ceroid lipopigments. The endoplasmic reticulum (ER) is a critical organelle for normal cell function. Alteration of ER homeostasis leads to accumulation of misfolded protein in the ER and to activation of the unfolded protein response. ER stress and the UPR have recently been linked to the NCLs. In this review, we will discuss the evidence for UPR activation in the NCLs, and address its connection to disease pathogenesis. Further understanding of ER-stress response involvement in the NCLs may encourage development of novel therapeutical agents targeting these pathogenic pathways.

## Highlights

• ER-stress activation has been linked to various neurodegenerative diseases.
• ER-stress is a common patho-mechanism in four forms of NCL.
• Pharmacological modulation of UPR could provide new treatment for NCL.

## 1 Introduction

The neuronal ceroid lipofuscinoses (NCLs), also known as Batten disease, represent a group of lysosomal storage disorders (LSD) with a worldwide distribution, affecting both children and adults. Thirteen genetically distinct genes have been identified that, when mutated, result in abnormal lysosomal function and an excessive accumulation of ceroid lipofuscin in neurons and other cell types. The NCL family of proteins is comprised of four lysosomal enzymes (PPT1/CLN1, TPP1/CLN2, CTSD/CLN10, CTSF/CLN13), a soluble lysosomal protein (CLN5), secreted proteins (GRN/CLN11), and several proteins that display different subcellular localisations (CLN3, DNAJC5/CLN4, CLN6, MFSD8/CLN7, CLN8, ATP13A2/CLN12, KCTD7/CLN14). Unfortunately, the precise function and localisation of many of the NCL proteins are still unclear, which has made targeted therapy development challenging. In this review we present and discuss the pathogenic role of ER stress in the listed NCL subtypes.

### 1.1 Neuronal ceroid lipofuscinoses

The NCLs are the most common autosomal recessive neurodegenerative storage disorders, and have no effective treatment. The hallmark characteristic is the presence of autofluorescent storage material in lysosomes resembling ceroid and lipofuscin lipopigments that also accumulate in disease or during the normal ageing process. This storage material is observed in many tissues and cell types. However, the pathological effect of the disease is most prominent in the central nervous system (CNS) and the eyes. There are several types of NCLs, each of which is caused by mutation in a distinct gene and differ for age of onset, disease progression and severity, and the morphological appearance of the storage material [1]. Historically, classification of the NCLs was clinically led according to the age at onset of symptoms: infantile, late infantile (LINCL), juvenile (JNCL) and adult (ANCL) NCLs. However, the NCLs are more genetically heterogeneous than initially thought. Mutations in the same gene can lead to very different disease courses [2]. An internationally developed new NCL nomenclature now clearly identifies each NCL disease both genetically and clinically [1], [3] with diagnosis now highlighting the defective gene as well as the age at disease onset. To date, more than a dozen different genes have been found to cause NCL [2], [4]. Common pathological characteristics of NCL include progressive visual deterioration leading to blindness, epilepsy, intellectual and motor decline, with affected individuals dying prematurely [1]. The ultrastructural appearance of the storage material is characteristic for different types of NCL and has long been used as a diagnostic tool [1]. Intracellular localisation and function of the identified NCL proteins vary: five NCL types are caused by defects in soluble lysosomal enzymes or proteins (CLN1; CLN2; CLN5; CLN10; CLN13), other forms by defects in transmembrane proteins with various localisations (CLN3; CLN6; CLN7; CLN8; CLN12; CLN14) [2], [4]. In addition, the recently identified *CLN4* (*DNAJC5*) and *CLN11* (*GRN*) genes encode, respectively, a cytosolic and extracellular proteins [2], [4] (Table 1). How all these genetic defects lead to similar clinical symptoms and neurodegeneration is still not clear.

**Table 1:** Summary of the identified genes causing human NCL disease. In bold are the genes linked to ER stress discussed in this review.

Gene	Protein	Protein solubility	Protein localisation	Linked to ER stress	Refs
***CLN1***	**PPT1** **Palmitoyl protein thioesterase1**	**Soluble**	**Lysosomal matrix**	**Yes**	[27], [60], [62], [63], [66]
*CLN2*	TPP1 Tripeptidyl peptidase1	Soluble	Lysosomal matrix	Unknown	
***CLN3***	**CLN3**	**Transmembrane**	**Golgi/ Lysosome**	**Yes**	[75], [76], [77], [78], [80], [81]
*CLN4*	DNAJC5	Soluble	Cytosolic, associated with vesicular membrane,	Unknown	
*CLN5*	CLN5	Soluble	Lysosomal matrix	Unknown	
***CLN6***	**CLN6**	**Transmembrane**	**ER-membrane**	**Yes**	[86], [88], [89]
*CLN7*	MFSD8 Major facilitator superfamily domain-containing protein-8	Transmembrane	Lysosomal membrane	Unknown	
***CLN8***	**CLN8**	**Transmembrane**	**ER-membrane**	**Yes**	[86], [93], [98], [99], [100], [101]
*CLN10*	CTSD Cathepsin D	Soluble	Lysosomal matrix	Unknown	
*CLN11*	GRNProgranulin and granulins	Soluble	Extracellular	Unknown	
*CLN12*	ATP13A2	Transmembrane	Lysosomal membrane	Unknown	
*CLN13*	CTSF Cathepsin F	Soluble	Lysosomal matrix	Unknown	
*CLN14*	KCTD7	Transmembrane	Partially associated with plasma membrane	Unknown	

^⁎^*CLN9* gene has been postulated but not yet identified [104].

### 1.2 ER-Golgi transport

Proteins destined for secretion, membranes or organelles begin their maturation in the ER where they fold, oligomerise, and are frequently subjected to covalent modifications in the process of becoming biologically active. The secretory pathway comprises the endoplasmic reticulum (ER), ER exit sites (ERES), the ER-to-Golgi intermediate compartment (ERGIC), the Golgi complex and post-Golgi carriers en route to their final destination. To exit the ER, newly synthesised proteins must pass a tightly controlled quality check [5]. Proteins that fail to do so are rerouted to the cytosol for degradation. It is important to understand the molecular mechanisms that coordinate protein synthesis, folding, transport and degradation because unbalances in these processes are the basis of many human diseases. The mechanisms that direct ER-Golgi cargo flow involve specialised multi-protein machineries. The three main vesicular frameworks are COPII, COPI and clathrin-based.

COPII-coated vesicles transport cargo proteins from the ER to the Golgi; COPI-coated vesicles transport cargo in the retrograde direction (from the cis-Golgi back to the ER) and between Golgi cisternae. Clathrin-coated vesicles form at the plasma membrane and the trans-Golgi network (TGN) and subsequently fuse with endosomes or lysosomes. By coupling cargo selection to vesicle formation, cells can achieve efficient protein sorting as an in-built outcome of the transport pathway itself. In addition, some cargoes exit the ER without concentration into vesicles or interaction with the vesicle coat [6].

Understanding how protein-folding influences ER–Golgi traffic is crucial, since premature exit from the ER results in a lack of folding and of access to the degradation machinery. Cargo adaptors and cargo receptors in both the ER and Golgi could play direct roles in determining the fidelity of transport. ER receptors discriminate between fully folded proteins and those that need further chaperone action; Golgi receptors bind misfolded cargo for retrieval back to the ER. However, whilst this general role may suffice for some misfolded proteins it is unlikely to apply to all proteins. Many incompletely folded proteins are actually transport-competent beyond the ER [7]. This may be because some ER export signals are simple peptides extending from the cytoplasmic side of the membrane and it is unlikely that their recognition is strongly influenced by protein folding on the luminal side of the membrane. Therefore, some misfolded proteins may be recognised as normal by the export machinery. It is more accurate to describe the ER exiting process as a dynamic event under the control of multiple competing interactions, such as ER export and retention signals and ER associated degradation (ERAD). Furthermore, under severe ER stress condition, sorting signals can be used as an ER detoxification pathway [8]. Understanding the interplay between these pathways lies at the heart of numerous misfolding diseases that stem from defects in quality control of protein sorting at the ER–Golgi interface.

### 1.3 Endoplasmic Reticulum (ER) stress response

The ER is a complex, dynamic organelle and is the first compartment in the secretory pathway responsible for the synthesis, modification and export of newly synthesised proteins. The ER is also involved in Ca^2+^ storage, lipid and carbohydrate metabolism. Cellular stress, induced by internal or external cues, activates several orchestrated processes aimed at either restoring cellular homeostasis (adaptive UPR) or committing to cell death (maladaptive UPR). Those processes named ‘ER stress response’ comprise various aspects such as unfolded protein response activation, autophagy, and mitochondrial response. When one of the elements of the ER stress response is impaired, as often occurs under pathological conditions, overall cellular homeostasis may be perturbed [9]. The quality control mechanism of the ER is therefore essential to ensure that only properly folded proteins exit the ER.

#### 1.3.1 The unfolded protein response (UPR)

The overall protein folding status is relayed to the cytosol, nucleus and other organelles such as lysosomes and mitochondria. During times of ER stress, protein load and translation are decreased and the folding capacity of the ER increased in an attempt to restore the ER equilibrium. To reduce the protein load, transcription of genes encoding secretory proteins is downregulated, misfolded proteins are cleared through ERAD, and a general reduction of protein translation is imposed at the ER. More chaperones are synthesised and the volume of the ER is increased in order to dilute the unfolded protein load and to host more chaperone machineries in an attempt to increase the protein folding capacity. If the aforementioned mechanisms fail to rescue and restore cell homeostasis then apoptosis could be triggered through the intrinsic pathway in an attempt to prevent further damage at the level of tissue or organism [9].

The protein folding machinery of the ER consists of three classes of proteins, foldases, molecular chaperones and lectins. Foldases directly promote protein folding and molecular chaperones prevent protein aggregation by shielding unfolded regions. The ER-resident chaperones comprise the HSP70 family, GRP78, LHS1/GRP170, their co-chaperones, which belong to the DnaJ and GrpE families, and an HSP90 paralogue, HSP90B1, which recognises partially folded proteins [10]. Lectins function as chaperone and sorting receptors in the secretory pathway. Calnexin and calreticulin are ER-specific lectins that recognise terminal glucose residues on high-mannose N-linked glycans [11]. Prolonged interaction of a native protein with the chaperone machinery is a key step in inducing the unfolded protein response.

**Fig. 1:**
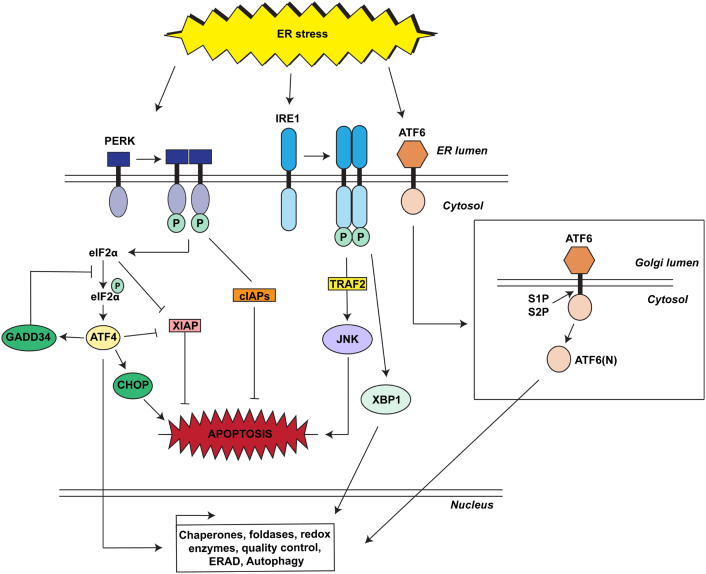
Schematic view of UPR response in mammals. Both survival and pro-apoptotic pathways are depicted in the figure. Upon disruption of ER homeostasis, three ER stress sensor proteins (PERK, IRE1and ATF6) and their specific signalling cascades are activated leading to expression of target genes encoding ER chaperones, foldases, redox enzymes and proteins involved in ER associated degradation (ERAD) and/or autophagy. When these adaptive responses are not sufficient to resolve the ER stress, the maladaptive pathway of UPR is activated, leading to apoptosis.

Three ER membrane sensors regulate the UPR: inositol-requiring enzyme 1 (IRE1), activating transcription factor 6 (ATF6) and double-stranded RNA-activated protein kinase-like ER kinase (PERK), all of which can be activated by perturbed ER homeostasis. Each activates specialised transcriptional programmes mediated by distinct transducers [9]. Two forms of IRE1 are present in mammals: IRE1α is expressed ubiquitously while IRE1β is expressed only in the intestinal and lung epithelium [12]. The transcription factor downstream of IRE1α, XBP1, activates target genes involved in protein folding and the major chaperones BiP/GRP78 and GRP94 [13]. IRE1 is also linked to apoptosis via activation of the Jun/JNK pathway [14]. ATF6 is an ER transmembrane protein that is internalised and transported to the Golgi complex upon ER stress. Once in the Golgi ATF6 is cleaved by Site-1 protease (S1P) and Site-2 protease (S2P), liberating the N-terminal cytosolic fragment, ATF6(N), which translocates to the nucleus to activate UPR target genes [15]. Interestingly, there is a relationship between S1P and lysosomal function. Absence of S1P results in lack of cleavage and activation of the α/β-subunit precursor of the hexameric *N*-acetylglucosamine-1-phosphotransferase complex leading to abnormal sorting of newly synthesised lysosomal proteins and accumulation of storage material in the lysosomes, a similar phenotype to that observed in mucolipidosis type II (MLII) [16]. ATF6(N) targets genes involved in protein folding such as BiP/GRP78, protein disulphide isomerase (PDI) and glucose-regulated protein 94 (GRP94), and protein degradation such as components of the ERAD [15]. Increased expression of molecular chaperones and of degradation enzymes helps to relieve the ER from the stress (Fig. 1).

PERK, an ER transmembrane kinase, functions as an ER-stress sensor, influencing redox homeostasis and the ER-mitochondria interface [17], [18]. Upon ER stress, PERK oligomerises, auto-phosphorylates, and phosphorylates the α subunit of the eukaryotic initiation factor 2 (eIF2α) (Fig. 1). eIF2α phosphorylation leads to a temporary attenuation of overall protein translation and to an upregulation of the transcription factor ATF4. This translation inhibition decreases the load of proteins entering the ER in an attempt to alleviate the ER stress and therefore playing an adapting role in direct response to ER stress. In addition, the PERK pathway plays a master and dual role in apoptosis. Increased expression of the transcription factor ATF4 upregulates C/EBP homologous protein (CHOP) and DNA damage-inducible 34 (GADD34) (Fig.1) [19]. CHOP promotes ER stress-induced apoptosis and GADD34 is involved in a negative feedback loop that counteracts PERK by dephosphorylation of eIF2α, allowing protein synthesis to resume and triggering apoptosis (Fig. 1) [20]. In addition, activation of PERK can lead to downregulation of anti-apoptotic protein XIAP, through eIF2α and ATF4, causing an increase in apoptosis [21] (Fig. 1). However, PERK also functions in preventing ER-stress induced apoptosis. PERK activates the cytoplasmic inhibitors of apoptosis proteins (cIAPs), regulating the cross talk that influences UPR determination of cell fate under ER stress (Fig. 1) [22]. In addition, PERK is required to regulate inter-organelle cross talk in reactive oxidative species-induced cell death (paragraph 1.3.2) and is a molecular mediator of the ER-mitochondria contact sites (paragraph 1.3.4) [18]. It is still unclear if in this context PERK is acting as a UPR mediator or independently from the ER stress response [23].

#### 1.3.2 ER stress and apoptosis

Apoptosis is a highly sophisticated and regulated programme that requires activation of different molecular events. Studies indicate that there are two main apoptotic pathways: extrinsic (death receptor pathway) and intrinsic (mitochondrial pathway). The extrinsic pathway is triggered by transmembrane receptors belonging to the tumor necrosis factor (TNF) superfamily. These receptors contain a cytoplasmic “death domain” that transmits a signal from the cell surface into the cell, activating a signal cascade that culminates in activation of caspase 8. Activated caspase 8 cleaves downstream substrates including other caspases, thereby activating the execution phase of the apoptotic process [24]. The intrinsic pathway can be triggered by different signals resulting in alteration of the mitochondria membrane potential and release of pro-apoptotic factors. Apoptotic and survival pathways are tightly controlled and regulated. To counterbalance the apoptotic pathways, cells express a variety of anti-apoptotic factors that counteract cell degeneration caused by both environmental and genetic insults [25]. The fine tuning between cell survival and death is regulated by members of the Bcl-2 family that consist of both anti-apoptotic proteins such as Bcl-2 and Bcl-xL and pro-apoptotic factors such as Bax, Bak, Bik and Bim [24]. The anti-apoptotic factor Bcl-2 controls the integrity of the mitochondrial membrane under normal conditions [24]. The pro-apoptotic protein Bik largely localises to the ER, whereas Bim translocates to the ER membrane and is important for ER stress-mediated cell death [26].

In addition, accumulation of a protein called growth-associated protein 43 (GAP43) in the ER is also able to trigger ER-dependent apoptosis [27]. GAP43 is a highly palmitoylated protein mainly expressed in neurons, and an increase in its mRNA has been detected in patients with amyotrophic lateral sclerosis [28]. Abnormal accumulation of GAP43 within the ER causes stress and UPR activation [27] and activation of ER-resident caspase 4 [27] leading to apoptosis through activation of caspase 3 and subsequent cleavage of the apoptotic effector PARP. Alternatively, the apoptotic pathway can be activated through elevated production of reactive oxygen species (ROS), through the role of PERK as ER-mitochondria mediator [18], [27]. ROS production causes release of Ca^2+^ ions, which destabilise mitochondrial membranes and activating caspase 9 with subsequent activation of caspase 3 and cleavage of PARP leading to cell death [27].

In addition, IRE1 pathway activation can lead to Jun/JNK mediated apoptosis [14]. Upon prolonged stress, the cytoplasmic domain of IRE1 can interact with TRAF2, reflecting in a phosphorylation of JNK and activation of the apoptotic pathway. Conversely, JNK-induced apoptosis can amplify the IRE1 signal, via pro-apoptotic factors such as Bax and Bak [29].

Apoptosis under ER stress is therefore the result of fine-tuning between different branches of the UPR pathway. Whether or not ER stress culminates in cell death is determined by multiple different factors, such as the intensity and duration of the stress. Evidence for a role of ER stress-mediated apoptosis in different neurodegenerative diseases make this process an attractive target for therapy. Thus, a better understanding of the mechanisms that orchestrate the ER stress mediated apoptosis may help to shape future therapeutic strategies.

#### 1.3.3 ER stress and autophagy

Accumulating data indicate that ER stress is also a potent trigger of autophagy [30]. Autophagy is a lysosomal pathway involved in the turnover of cellular macromolecules and organelles. The first step of autophagy is the envelopment of cytosol and/or organelles by the isolating membrane, which wraps around the cargo forming an autophagosome, a vesicle surrounded by a double-membrane. The autophagosome then undergoes progressive maturation following fusion with endolysosomal vesicles to create an autolysosome, where the cargo is degraded. Autophagy is controlled by a set of evolutionarily conserved autophagy-related proteins (Atg proteins) [31]. The initial nucleation and assembly of the primary autophagosomal membrane requires a kinase complex that consists of class III phosphatidylinositol 3-kinase (PI3K), p150 myristoylated protein kinase and beclin 1 (also known as Atg6). Further elongation of the isolating membrane is mediated by two ubiquitin-like conjugation systems one of which results in conversion of microtubule-associated protein 1 light chain 3 (LC3; also known as Atg8) from free form (LC3-I) to a lipid-conjugated membrane-bound form (LC3-II). The accumulation of LC3-II and its localization to vesicular structures are commonly used as markers of autophagy [31]. Autophagy is also involved in removing damaged or excess organelles. For example, mitochondria that have lost their membrane potential and peroxisomes changing in response to environmental cues can be selectively removed by autophagy [31].

Whereas the induction of autophagy by ER stress is conserved from yeast to mammals, the signalling pathways responsible for autophagy induction and its cellular consequences appear to vary according to the cell type and the stimulus. Recently, it has been shown that the transcription factors TFE3 and TFEB [32] contribute to UPR and autophagy. It has been suggested that TFEB and TFE3 may play a dual role in determining cellular fate. During early stages of ER stress or cellular starvation, TFEB and TFE3 may promote cell survival by enhancing expression of genes such as ATF4, promoting expression of genes required for autophagy, lysosomal biogenesis, and ER homeostasis. Under prolonged stress, instead, activation of TFEB and TFE3 leads to ATF4 dependent expression of pro-apoptotic factors [33].

A better understanding of the signalling pathways controlling autophagy and the cellular fate in response to ER stress will hopefully open new possibilities for the treatment of the numerous diseases related to ER stress.

#### 1.3.4 ER stress and mitochondria

Many regulatory members of UPR link with mitochondrial regulation and function [34]. The ER and mitochondria are physically and functionally connected through mitochondria-associated ER membranes (MAMs). The MAMs are crucial for the regulation of Ca^2+^ homeostasis in response to a specific signalling pathway. PERK is enriched at the ER side of the MAMs where it plays an important role in integrity [18] and is a regulator of mitochondrial processes such as fission and fusion [35]. Despite the function of PERK as ER-mitochondria regulator appearing independent of its role in the UPR, an UPR-dependent role cannot be excluded [23]. In addition, ATF4, another member of the UPR response, controls expression of the ubiquitin kinase Parkin, a key player of mitochondrial function and dynamics [36]. Parkin has also been increasingly reported in MAM, where it maintains ER-mitochondrial Ca^2+^ transfer [37]. In addition, Parkin is also able to ultimately regulate XBP1 level, modulating therefore various UPR dependent responses [38].

Moreover, in skeletal muscle ATF6 is an important partner of PCG1 (peroxisome proliferator-activator receptor gamma coactivator 1 alpha), which is associated with mitochondrial biogenesis [39].

The link between ER and mitochondrial stress is still under debate even though defects in this axis have been linked to various neurodegenerative disease [40]. Further studies on this topic will further understanding of the pathophysiology and the identification of potential therapeutic treatment.

### 1.4 ER stress and UPR in neurodegenerative disorders

A common feature of many neurodegenerative disorders is the accumulation and deposit of misfolded and unfolded proteins, which trigger ER stress affecting neuronal homeostasis and lead to cell death [41]. The ER stress response underlies numerous adult and paediatric neurodegenerative diseases such as Parkinson's disease (PD) [42], Alzheimer's disease (AD) [43], amyotrophic lateral sclerosis [44], polyglutamine diseases [45], as well as paediatric disorders such as lysosomal storage diseases (LSD) and hereditary spastic paraplegia (HSP) [46].

In Parkinson's disease, ER stress together with abnormal protein degradation plays an important role in the pathophysiology of PD, contributing to the selective death of dopaminergic neurons [42]. Similarly, in Alzheimer's disease ER stress and altered Ca^2+^ homeostasis [47] have been demonstrated to be part of the etio-pathogenesis of the disease. However, ER stress activation is not a distinctive feature of PD and AD. Indeed, in amyotrophic lateral sclerosis elevated levels of ROS and Ca^2+^ caused by accumulation of toxic products are able to trigger ER stress eventually leading to motor neuron loss [44]. In addition, expanded polyglutamine (PolyQ) repeats in different proteins can cause inherited neurodegenerative disease such as: Huntington's disease, spinobulbar muscular atrophy, dentatorubral-pallidoluysian atrophy and at least six spinocerebellar ataxias. These diseases are characterized by accumulation of intracellular protein aggregates, ER stress and selective neuronal death [45].

ER stress has been shown to play a role also in the pathophysiology of paediatric diseases such as hereditary spastic paraplegia (HSP), an infantile inherited autosomal dominant neurological disorder characterized by spastic weakness of the lower extremities [46], and LSD. There are at least 50 different disorders classified as paediatric LSD, including: G_M1_-gangliosidosis, Pelizaeus-Merzbacher disease (PMD) and the NCLs. Evidence suggests that ER stress and the UPR response accompany G_M1_-gangliosidosis [48] and Pelizaeus-Merzbacher disease (PMD) [49]. These diseases are characterized respectively by accumulation of G_M1_-ganglioside [48] and hypomyelination of the central nervous system [49].

In addition, various reports suggested a link between ER stress and four forms of NCL disease (CLN1, CLN3, CLN6 and CLN8) (Table 1). For the remaining genes involved in the NCLs a link with ER stress is less clear. No link with ER stress has so far been demonstrated for five NCL genes (CLN2, CLN5, CLN7, CLN12 and CLN14), although investigations have not been exhaustive. However, for four other NCL subtypes (CLN4, CLN10, CLN11 and CLN13) a correlation with the ER stress could be hypothesised based on existing observations, although no formal evidence is available yet. Mutations in the *CLN4* gene cause the adult onset form of NCL known as Kufs disease. *CLN4* or *DNAJC5* encodes cysteine-string protein α (CSPα). CSPα is a chaperone that ensures protein folding [50]. Mutations in CSPα could cause accumulation of unfolded presynaptic proteins that then trigger UPR. *CLN11/GRN* (progranulin) has been suggested to play a role in lysosomes [51] and autophagy under nutrient-starvation, decreasing mTORC1 and TFEB [52], [53], therefore suggesting a potential link to ER stress. Mutations in *CLN13* (*CTSF*) [54] and *CLN10* (*CTSD*) have been tightly related to protein degradation, apoptosis and autophagy [55], also hinting at a potential role of ER stress in the pathogenesis.

**Fig. 2:**
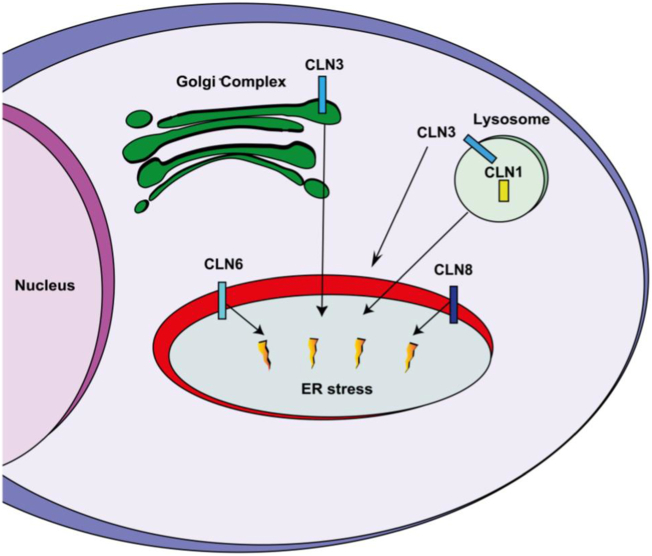
Mutations in three transmembrane proteins (CLN3, CLN6 and CLN8) and one lysosomal enzyme (CLN1) cause distinct types of NCL that include activation of ER stress, eventually leading to apoptosis.

As mentioned earlier, a strong correlation between ER stress and the disease gene has been described in four NCLs subtypes: CLN1, CLN3, CLN6 and CLN8 diseases. We now review the pathogenetic role of ER stress in these listed NCL subtypes (Fig. 2).

## 2 NCL proteins and ER stress

### 2.1 CLN1/PPT1

Most cases of infantile NCL are caused by inactivating mutations in palmitoyl-protein thioestarase-1 (PPT1), encoded by *PPT1/CLN1* [56]. PPT1 is a lysosomal protein that catalyses the cleavage of thioester linkages in reversible S-acylated (palmitoylated) proteins, facilitating the degradation and/or recycling of these lipid-modified proteins [57]. Children affected by classic CLN1 disease manifest symptoms at 6–12 months, with brain atrophy causing cognitive and motor impairment by 2 years of age and death by 10–12 years of age [58]. However, some mutations cause a more prolonged disease of later onset such as juvenile and adult CLN1 disease [59].

It has been reported that some PPT1 variants containing disease-causing mutations are not correctly transported to the lysosomes, but are retained in the ER due to improper folding [60] where they are presumably degraded. For others, transport to the lysosome may still occur but the enzyme activity is abolished due to loss of the catalytic site. Moreover, elevated neuronal levels of apoptosis have been reported in both brain biopsy [61] and in cultured cells from CLN1 patients [62]. Apoptosis, in CLN1 disease, has been proposed to be caused by ER stress-mediated caspase activation [27], [63] as well as production of ROS that cause Ca^2+^ dyshomeostasis with activation of caspase 9 and 3 [27]. Tissues from mice deficient in PPT1 have abnormal ER morphology, activation of UPR and undergo apoptosis via activation of caspase 3 [64]. These observations suggest that apoptosis arising from ER stress is a major cause of cell death in CLN1 disease. In addition, treatment with a chemical chaperone such as trimethylamine *N*-oxide dihydride (TMAO) or tauroursodeoxycholic acid (TUDCA) [65] significantly decreases apoptosis in cells from CLN1 patients suggesting that UPR may be a primary target for CLN1 disease treatment [66]. Exactly how decreased or absent PPT1 activity in the lysosome causes ER stress remains to be understood. It may be due to lysosomal-ER cross talk that directly connects the consequences of aberrant lysosomal depalmitoylation with the ER, or a consequence of the more general disruption of lysosomal homeostasis on the cell that disturbs normal ER function. Regardless, mutations in PPT1 lead to activation of ER stress and UPR and may provide targets for therapeutic development.

### 2.2 CLN3

The majority of juvenile cases of NCL, with an age of onset ranging between 5 and 7 years of age, are caused by mutations in the *CLN3* gene. More than 40 disease-causing mutations have been identified in *CLN3* (http://www.ucl.ac.uk/ncl) [4] however the majority of CLN3 disease patients carry a 1 kb deletion [1] with around 74% homozygous for this mutation and about 22% compound heterozygous for this and another rarer mutation [67]. The 1 kb deletion in *CLN3* results in multiple transcripts that include a frame-shift and premature termination or loss of several internal exons and an in-frame C terminus and retained protein function [68]. There are also studies showing that mutant transcripts of CLN3 are targeted for nonsense decay, and absence of mutated proteins [69], [70]. *CLN3* encodes a 438 amino acid transmembrane protein and despite being identified more than two decades ago, the function of this highly conserved protein remain elusive. Levels of endogenous CLN3 protein are extremely low. A significant localisation of the CLN3 orthologue of fission and budding yeasts, Btn1p, has been shown at the Golgi complex [71] and also reported for GFP-tagged CLN3 in mammalian cells [72]. Moreover, untagged CLN3 has been shown to localise at late-endosomes and lysosomes [73]. However, the functional localisation of CLN3 is still matter of debate. Although, the function of CLN3 remains unknown, a variety of studies have shown that CLN3 influences many cellular processes including protein trafficking [74], lysosomal homeostasis [75] mitochondrial functions [76] Ca^2+^ homeostasis and autophagy [77]. CLN3 disease neuropathology is associated with excessive neuronal and photoreceptor cell apoptosis [78]. In addition, previous study has suggested that *CLN3* is an anti-apoptotic gene in neuronal precursor cells and in at least some cancers [79]. When using *Drosophila* as a CLN3 disease model, ER and mitochondrial functions were found to be impaired by oxidative stress [80]. Furthermore, the apoptosis leading to the neurodegeneration central to CLN3 disease may be caused by improper management of the response to ER stress [81]. Indeed, it has been shown that overexpression of wild-type CLN3 enhanced expression of the ER chaperone protein, glucose-regulated protein 78 (GRP78), and reduced expression of the proapoptotic protein CCAAT/-enhancer-binding protein homologous protein [81]. In contrast, overexpression of mutant CLN3 or siRNA knockdown of CLN3 produced the opposite effect. Together, the evidence suggests that lack of CLN3 function in cells leads to a failure of management of the response to ER stress [81].

Recently, it has been shown that treatment of lymphoblast cells from patients with fibrates, drugs able to induce peroxisome proliferator-activated receptor-α (PPAR-α) activation, abrogates the depolarization of mitochondrial membrane potential, normalises defective autophagy and increases overall cell viability [82].

Together, the evidences so far therefore strongly suggest that ER stress activation may play a pivotal role in the pathogenesis of CLN3 disease, and certainly this requires further study.

### 2.3 CLN6

There is little current understanding of how mutations in *CLN6* cause CLN6 disease [83], which is typically of late infantile onset but may also arise in adulthood [84]. CLN6 is a 311 amino acid transmembrane ER protein (27 kDa) of unknown function and with no conserved homologous proteins [83], [85]. The function of CLN6 has been associated with autophagy, regulation of pH and endocytosis [86]. CLN6 is retained in the ER with no observed localisation to the lysosome and no effect on the synthesis, sorting nor proteolytic processing of the lysosomal enzyme cathepsin D in fibroblasts from patients with CLN6 disease, sheep (OCL6) or mouse (*nclf*) models [87]. However, the lysosomal enzyme arylsulfatase A responsible for the lysosomal hydrolysis of sulfatides, is reduced in mutant CLN6 cell lines [87].

Accumulation of biometals such as zinc, copper, manganese and cobalt has been described in Merino and South Hampshire sheep models of CLN6 disease [88], [89], as well as in the spinal cord, brain cortex, heart and liver of a Cln6^nclf^ mouse disease model which carries a 1 bp insertion in the orthologous gene [90]. Aberrant biometal metabolism is a common feature observed in other neurodegenerative disorders such as Alzheimer's and Parkinson's disease [91]. Further research efforts are needed to clarify the precise function of CLN6. Nevertheless, the selective neurodegeneration arising from CLN6 deficiency could be linked to this accumulation of biometals, especially to zinc. Zinc is a key modulator of intracellular and intercellular neuronal signalling, and zinc dyshomeostasis can cause neuronal cell death. Substantial progressive loss of the ER/Golgi-resident Zn transporter, Zip7, which colocalises with CLN6, may contribute to the subcellular deregulation of biometal homeostasis in NCL disease. Importantly, the metal-complex Zn^II^(atsm) that is protective in amyotrophic lateral sclerosis-induced Zip7 upregulation, promotes Zn redistribution and restores Zn-dependent functions in primary mouse *Cln6* deficient neurons and astrocytes [89]. Both ER and oxidative stress have been observed in CLN6 disease [88]. It is possible that biometal dyshomeostasis impairs processing of protein folding, causing activation of ER stress with subsequent apoptosis. In addition, it has been reported that TRAM1, a protein involved in translocation of nascent polypeptides during ER stress, is also upregulated during UPR causing the rapid degradation of CLN6 mutants [92]. Altogether, this evidence implicates ER quality control in the stability of CLN6 variants and a likely contribution to the pathogenesis of CLN6 disease.

### 2.4 CLN8

*CLN8* is mutated in two distinct clinical NCL phenotypes: late infantile and a mutation-specific juvenile-onset variant, progressive epilepsy with mental retardation (EPMR) also known as Northern Epilepsy [93]. Like CLN6, CLN8 is an ER-resident transmembrane protein of predicted 286 amino acid residues containing 5 hydrophobic regions including a TLC (TRAM-LAG1-CLN8) domain. It is recycled between the ER and ER- Golgi intermediate compartment (ERGIC) using an ER-retrieval signal (KKRP) in its cytoplasmic C-terminus in non-neuronal cells [94], [95], [96]. Mutations in CLN8 do not appear to affect its localization at the ER but it has been observed outside the ER in a specialised sub-compartment of the polarised epithelial Caco-2 cells [97], in the Golgi, endosomes and lipid rafts of mouse fibroblasts, in a light membrane fraction different from ER after fractionation of mouse brain, as well as in the proximity of the plasma membrane in hippocampal neurons. Although not delineated, the function of CLN8 has been linked to lipid homeostasis [86]. This may provide a link between CLN8 mutants and ER stress, as the UPR can be regulated by lipids and also plays a role in regulating lipid metabolism [98]. ER stress may therefore play a crucial role in the pathogenesis of the disease. BiP/GRP78, CHOP, and ATF6 are upregulated with activation of caspase 12 in the CNS and retina of the naturally occurring *Cln8*^mnd^ mouse model, which exhibits a disease phenotype similar to that of late-infantile CLN8 disease patients [93], [99]. In addition in *mnd* mice defective lipids and protein transport and ER/mitochondria Ca^2+^ exchange are linked to alteration in MAMs [100].

Recently, molecular partners of CLN8 have been described to be involved in autophagy, apoptosis, as well as vesicular trafficking and lipid transport [101]. All this evidence together therefore strongly suggests that apoptosis induced by ER stress activation may play a pivotal role in the pathogenesis of CLN8 disease, and certainly deserves further study.

## 3 Conclusions

Experimental evidences suggest that several organelles are capable of sensing and relaying pro-apoptotic signals, culminating in the proteolytic activation of caspases and cell death. Each organelle is likely to be uniquely poised and equipped to sense specific stimuli related to their function and structure. For example, the ER is the major site for folding, modification and assembly of newly synthesised proteins and has evolved a stress response pathway to cope with the accumulation of unfolded or misfolded proteins. Activation of ER stress has been shown to induce the expression of chaperones, attenuate translation and degrade misfolded proteins in attempt to alleviate the stress. Prolonged ER stress results in irreparable damage leading to apoptosis. This is the case observed in some of the NCL types described in this review (Fig. 2). Indeed, a mismanagement of the ER stress response leading to apoptosis is detectable in several types of NCLs (CLN1, CLN3, CLN6 and CLN8), with common pathways affected such as protein folding and build-up, ROS production, and caspase activation.

In CLN1 disease, both ROS and Ca^2+^ dyshomeostasis contribute to ER stress activation. In CLN3 disease, ER-stress mediated apoptosis might play an important role. A global screen to identify genetic interactions with the fission yeast orthologue *btn1* highlighted a conserved key signalling hub in which multiple components functionally relate to this conserved disease gene [102]. This signalling hub includes two major mitogen-activated protein kinase (MAPK) cascades, and centres on the Tor kinase complexes TORC1 and TORC2. Yeast cells modelling CLN3 disease exhibit features consistent with dysfunction in the TORC pathways. Wild type yeast cells respond to a variety of stresses via interconnected signalling pathways restoring cell homeostasis and integrity. In contrast, cells lacking *btn1* are unable to respond to the applied stresses, although the pathways themselves are still functional [102]. Thus, for CLN3 disease at least, neuronal cell death could be triggered by the inability of key signalling pathways to be activated, such as a dysfunctional mTORC1 pathway causing apoptosis through suppression of Akt and consequent induction of the IRE1–JNK pathway [103].

In CLN6 disease in particular, accumulation of biometals could lead to ER-stress. Biometals such as Mn^2+^ are important cofactors for protein post-translational modification, with defects in manganese homeostasis leading to misfolding of proteins and their accumulation in the ER and ER stress. In CLN8 disease, a mismanagement of the ER stress response is linked with lipid homeostasis and alteration of MAMs.

To date, the evidences reported are strongly suggestive that the dramatic neuronal loss observed in NCLs is caused by ER stress-mediated apoptosis, making this process an attractive target for therapy. The apoptotic pathway is not one single linear path, but is a complex and articulated pathway that can be activated via different stimuli and branches of the UPR. Given the various localizations and functions of the different CLN proteins, we cannot exclude that their mutation challenges the quality control and activates the ER stress/UPR in slightly different ways, but still resulting in a similar apoptotic phenotype. In addition, some mutant proteins are transport competent but not functional once reaching their final destination. Therefore, a better understanding of the interplay between the various pathways leading to the apoptotic pathway and arising from defects in the quality control process as well as how some proteins may escape quality control in the ER could greatly contribute to elucidating the pathogenesis of the various NCL diseases and provide specific therapeutic approaches. In addition, it is possible that alteration in one NCL gene influences the expression and localization of other NCL proteins, thereby generating a complex scenario in which modulation of the phenotype of various NCL diseases is the result of the combined alteration of expression of different NCL proteins.

### 3.1 Implications for treatment

The UPR pathway represents a possible common target to treat multiple forms of NCL disease. Therapeutically, it is therefore crucial to understand the role of the UPR in the pathogenesis of the NCLs. In addition, the pharmacological modulation of UPR, either by pro-apoptotic or pro-survival signalling, could provide new treatment strategies not only for the NCLs but many diseases, including other neurodegenerative disorders, heart disease and cancer.

## Transparency document

Transparency document. Image 1
